# Acute esophageal necrosis following kidney transplantation

**DOI:** 10.25122/jml-2021-0024

**Published:** 2021

**Authors:** Paul Thomas Kroner, Razvan Chirila, Monica Roxana Purcarea, Laura Tribus, Hani Michel Wadei

**Affiliations:** 1. Department of Gastroenterology and Hepatology, Mayo Clinic, Florida, United States of America; 2. Department of Internal Medicine, Mayo Clinic, Florida, United States of America; 3. Department of Nephrology, Carol Davila Clinical Nephrology Hospital, Bucharest, Romania; 4. Department of Gastroenterology, Bucharest Emergency University Hospital, Bucharest, Romania; 5. Department of Gastroenterology, Carol Davila University of Medicine and Pharmacy, Bucharest, Romania; 6. Department of Transplant Nephrology, Mayo Clinic, Florida, United States of America

**Keywords:** black esophagus, transplantation, tacrolimus, CMV – cytomegalovirus, EGD – esophagogastroduodenoscopy, GI – gastrointestinal, POD – postoperative day, TPN – total parenteral nutrition

## Abstract

We are reporting a case of spontaneous acute esophageal necrosis “black esophagus” of unclear etiology in a kidney transplant recipient. A patient with end-stage renal disease due to IgA nephropathy received a deceased-donor kidney transplant. The surgical procedure was uneventful, without hemodynamic instability. He was started on alemtuzumab for immunosuppression induction followed by maintenance immunosuppression with intravenous methylprednisolone for 3 days, then oral prednisone, mycophenolate mofetil and tacrolimus (a target level between 8 and 10ng/ml) daily. On postoperative day (POD) 3, the patient started to develop significant gastro-intestinal symptoms: epigastric pain, dysphagia, odynophagia, eructation, pyrosis, nausea, and regurgitation of food contents. He was diagnosed with esophageal necrosis by upper endoscopy on postoperative day 4. We describe a successful treatment with supportive therapy and complete recovery despite receiving immunosuppressive therapy. To our knowledge, this case is one of the few reported cases of esophageal necrosis in kidney transplant recipients and the first case that was not associated with clinical risk factors.

## Introduction

Kidney transplantation is a high-risk surgical treatment for patients diagnosed with end-stage renal disease [1]. Immunosuppressive therapy is required as a lifelong therapy for these patients and is initiated at the time of surgery. Transplant recipients undergo significant hemodynamic changes post-operatively and are at high risk for long-term infectious complications [1]. Gastrointestinal (GI) complications are common after kidney transplantation and mainly manifested as a side effect of the immunosuppressive medications [2]. Nausea, vomiting and diarrhea are the most commonly reported side effects. Serious GI complications are rare and mainly related to viral and fungal infections due to immunosuppressive medications [1–2].

## Case Report

A 48-year-old Asian male with end-stage renal disease due to IgA nephropathy received a deceased-donor kidney transplant. Both donor and recipient had positive cytomegalovirus (CMV) IgG antibodies prior to transplantation. Intra-operatively, the patient had stable blood pressure and did not experience intra-operative hypotension. He received a single 30 mg dose of alemtuzumab for induction, followed by maintenance immunosuppression with intravenous methylprednisolone for 3 days, then oral prednisone 5 mg daily. After that, he received mycophenolate mofetil 1 g twice daily and tacrolimus with a target trough level between 8 and 10ng/ml. On the postoperative day (POD) 3, the patient started to develop severe hiccups followed by sharp chest and epigastric pain with postprandial worsening, dysphagia, odynophagia, eructation, pyrosis, nausea, and regurgitation of food contents. His vital signs were stable with systolic blood pressure in the 150–160 mmHg range, and no evidence of fever or evidence of gastrointestinal bleeding was seen. Laboratory tests revealed a stable normocytic normochromic anemia with a hemoglobin of 9.8 g/dL without evidence of leukocytosis or thrombocytopenia and a normal coagulation profile. Tacrolimus level was at 11.1 ng/ml, slightly above the target range, when the symptoms started. On POD 3, his serum creatinine level was 15.4 mg/dl; therefore, hemodialysis was initiated. However, despite dialysis, his symptoms progressed.

**Figure 1.**
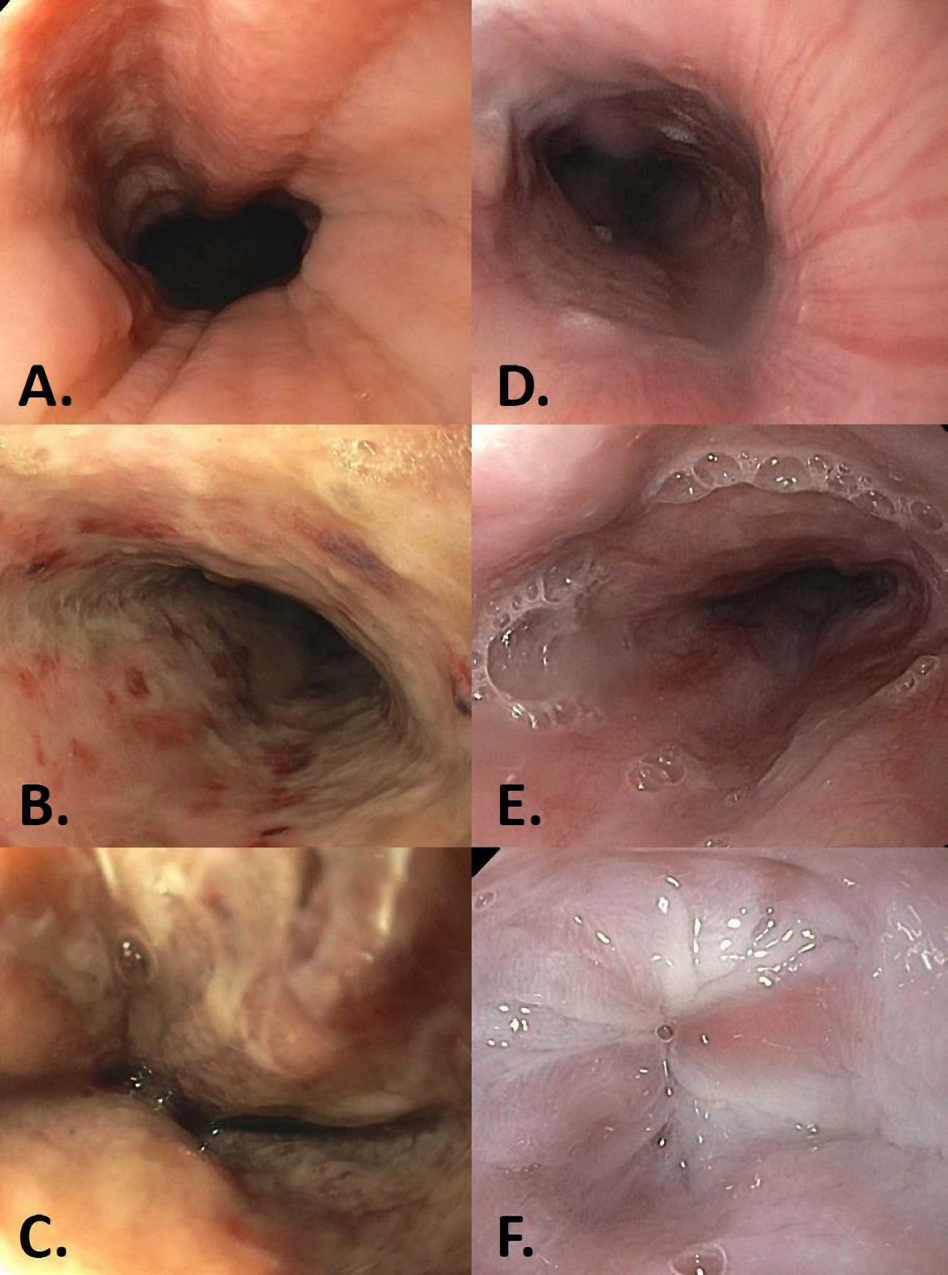
A) Normal-appearing upper esophagus on initial EGD; B) Middle esophageal tissue discoloration, erythema, and friability with contact bleeding; C) Lower esophageal tissue friability, ulceration and sloughing; D) Normal-appearing upper esophagus on follow-up EGD, one week afterward; E) Normal-appearing middle esophagus; F) Erythema and evidence of re-epithelization in the lower third of the esophagus.

On POD 4, the patient underwent esophagogastroduodenoscopy (EGD) that showed a normal upper esophagus (Figure 1A) with evidence of diffuse severe mucosal changes of the middle and lower third of the esophagus characterized by black-discoloration, erythema, and friable tissue with ulceration, sloughing and contact bleeding (Figure 1B-C). Biopsies were consistent with acute esophageal necrosis, also known as “black esophagus”. There was no evidence of viral inclusions on the surgical pathology specimen, so CMV infection was excluded. The gastric and duodenal examinations were normal. The patient’s oral intake was completely halted, was started on intravenous proton pump inhibitors, and was placed on total parenteral nutrition (TPN) for one week. Two weeks later, follow-up EGD revealed erythema in the lower third of the esophagus (Figure 1F), adequate re-epithelization, and no necrotic tissue visualization, consistent with recovery from an ischemic event (Figure 1D-F). By then, his symptoms had considerably improved, his diet was advanced, and he was discharged in a stable condition. One year after kidney transplantation, he reported no dyspepsia, odynophagia, or dysphagia. He had a stable serum creatinine level of 1.18 mg/dl with a glomerular filtration rate of 76 ml/min/1.73 m^2^.

## Discussion

Acute esophageal necrosis, known as “black esophagus”, is a rare complication visualized as a circumferential black-appearing esophagus typically found in the distal third, which has less vascular supply [3]. Related etiologies include hypoperfusion, infectious opportunistic pathogens (e.g., CMV, *Candida albicans*), diabetic ketoacidosis, malignancy, alcoholic hepatitis, or toxic ingestions [4]. Two cases of acute esophageal necrosis have been previously reported in kidney transplant recipients. In the first case, post-kidney transplant, acute esophageal necrosis was associated with intra-operative cardiac arrest, which resulted in hypotension and hypoperfusion to the distal esophagus. In the second case, acute esophageal necrosis was related to primary CMV infection. Other cases of acute esophageal necrosis have been reported in liver transplant recipients and have been associated with perioperative hemodynamic instability, such as cardiac arrest. It classically presents with hematemesis, melena, epigastric pain, or dysphagia [5]. In the absence of frank perforation, conservative management with proton pump inhibitors, halting of oral food and fluid intake, and follow-up EGD is the standard of care [6].

Our patient developed spontaneous acute esophageal necrosis that was not associated with an explicit hypoperfusion episode.

This case highlights that despite the reported association of acute esophageal necrosis with frank hypotension, infections, or uncontrolled diabetes, acute esophageal necrosis may occur in the setting of normal blood pressure and major surgery. Whether the slightly supratherapeutic tacrolimus level may have contributed to this potentially catastrophic complication is difficult to determine. However, tacrolimus has known vasoconstrictor properties in various vascular beds that could have potentially resulted in distal esophageal hypoperfusion and associated necrosis [7].

## Conclusion

The current case should raise clinician awareness in considering the possibility of esophageal necrosis in hemodynamically stable post-transplanted patients that develop sudden-onset symptoms of dysphagia and odynophagia, especially if a concomitant elevated tacrolimus level is present.

## Acknowledgements

### Conflict of interest

The authors declare that there is no conflict of interest.
